# Research on Relationship Among Internet-Addiction, Personality Traits and Mental Health of Urban Left-Behind Children

**DOI:** 10.5539/gjhs.v7n4p60

**Published:** 2014-12-16

**Authors:** Ying Ge, Jun Se, Jingfu Zhang

**Affiliations:** 1 Faculty of Psychology, Key Laboratory of Personality and Cognition, Ministry of Education, Southwest University, Beibei, Chongqing, China; 2 School of Education, Laboratory of Cognition and Mental Health, Chongqing University of Arts and Sciences, Yongchuan, Chongqing, China

**Keywords:** urban left-behind children, internet-addiction, personality traits, mental health

## Abstract

**Aim:**

In this research, we attempted at exploring the relationships among urban left-behind children’s internet-addiction, personality traits and mental health.

**Methods:**

In the form of three relevant questionnaires (Adolescent Pathological Internet Use Scale, Eysenck Personality Questionnaire, Children’s Edition in Chinese and Mental Health Test), 796 urban left-behind children in China were investigated, concerning internet-addiction, personality traits and mental health.

**Results:**

(1) The internet-addiction rate of urban left-behind children in China reached10.8%—a relatively high figure, with the rate among males higher than that among females. In terms of internet-addition salience, the figure of urban left-behind children was obviously higher than that of non-left-behind children. (2) In China, the personality deviation rate of the overall left-behind children was 15.36%; while the personality deviation rate of the internet-addicted urban left-behind children was 38.88%, a figure prominently higher than that of the non-addicted urban left-behind children group, with the rate among females higher than that among males. (3) The mental health problem rate of the overall urban left-behind children in China was 8.43%; while the rate of the internet-addicted urban left-behind children was 27.77%, a figure significantly higher than that of the non-addicted urban left-behind children. (4) There were significant relationships among internet-addiction, personality traits and mental health. The total score of internet-addiction and its related dimensions can serve as indicators of personality neuroticism, psychoticism and the total scores of mental health.

## 1. Introduction

As a brand new lifestyle, the internet plays a significant role in influencing people’s mentality and behavior. This is particularly true for adolescent group in China, which takes up 24.1% of the whole 618 million Chinese netizens (China Internet Network Development State Statistic Report, 2014). The negative outcome of human-internet interaction—internet-addiction, has thus become an increasingly prominent social problems. grabbing widespread attention from the society. Internet-addiction refers to an incontrollable online compulsion under no influence of addictive substances (Young, 1998). The cutting-off or decreased use of the internet may cause withdrawal reaction, accompanied with both physical and mental symptoms. In short, it is a gambling-like obsessive behavior (Young, 1998). In May, 2013, “internet-addition” was first officially included into *The Diagnostic and Statistical Manual of Mental Disorders* *(DSM-V)* (American Psychiatric Association, 2013).

Since China’s Reform and Opening-up, large number of migrant workers from the less developed western region came to economically prosperous and more developed eastern region for work and left their children at hometown, bringing about a newly emerged unique adolescent group: the left-behind children. Originally, the term “left-behind children” specifically referred to those children left in the rural area, who were either under the care of their grandparents, relatives and neighbors, or in independent life. Gradually, their mental health problems emerged and became increasingly prominent. Their personality traits were characterized by a detached parent-child relationship, anxiety, loneliness, indifference, etc (Duan & Zhou, 2005; Luo, Wang, & Gao, 2009; Zhao & Liu, 2010).

In recent years, of all the left-behind children, the rate of urban children begins to increase. According to the fifth population census, 13.5% of all the about 20 million left-behind children in China come from the urban areas (Lin, 2008). As the only municipality directly under the Central Government in western China, Chongqing boasts 1.07 million left-behind children, comprising 34% of the total students at compulsory education stage in this region (Education, Science, Culture and Public Heath Committee, Chongqing People’s Congress, 2012). The “urban left-behind children” refers to those children who are permanent urban residents under the age of 18, and have long been taken care of by nannies, grandparents, other relatives, or appointed non-relatives due to their both parents’ or one parent’s over half-year-long absence either for professional or academic reasons (Wang & Li, 2008; Zhou, Chen, & Deng, 2012). Generally speaking, this group of children is economically capable of accessing the internet. Without appropriate guidance on internet use, once attempted by complicated social environment, they easily fall victim to the internet, exhibiting deviation in personality and mental health. Currently, the incidence rate of internet-addiction in Chinese adolescents is between 6% and 14% (Zhen et al., 2009). As for adolescents in Chongqing, China, such incidence rate is 9.8% (Li, Wang & Xu, 2010).

Therefore, the purpose of this study is to investigate the internet-addiction extent of urban left-behind children, and to explore and analyze the personality traits and mental health status of their internet-addicted group.

## 2. Methods

### 2.1 Participants

With a combined approach of random sampling and purpose sampling, questionnaires were sent to 810 secondary school students selected both in downtown Chongqing and its suburban towns and districts, with 810 questionnaires returned, 796 of which were valid. Those selected students consisted of 401 male and 395 female students, 332 of whom were left-behind children.; while the remaining 464 of whom were non-left-behind children.

### 2.2 Tools

#### 2.2.1 Adolescent Pathological Internet Use Scale (APIUS)

*Adolescent Pathological Internet Use Scale* (APIUS) was made by Lei Li and Yang Yang (2007) and has been regarded as a proper tool to measure the internet-addiction of Chinese adolescent group. This APIUS consists of 38 questions in 6 dimensions, namely, salience—the use of internet as a decisive role in the customer’s thinking and behavior, mental alteration—the use of internet to change the customer’s negative mental state, social comfort—regarding online communication as a more comfortable, secure means of social contact, tolerance—the customer’s increased input of time and energy into the internet to obtain a sense of satisfaction, compulsive internet use—failure to achieve purposeful lessening of the time spent online, and exhibiting an obsessive fascination with the internet, and negative outcomes—the negative influence of internet use on normal life, with a focus on the internet-access related issues on interpersonal relationship, health and academic achievements. APIUS adopts 5-points based self-assessment scale, ranging from “definitely no” to “absolutely yes”. With KMO index of 0.940 and Cronbach’s Alpha coefficient of 0.948, this scale demonstrates high reliability and validity. According to APIUS, those whose average scores are equal or exceed 3.15 points are labeled as “internet-addicted group”, while those whose average scores are less than 3.15 points belong to “non-addicted group”.

#### 2.2.2 Eysenck Personality Questionnaire, Children’s Edition in Chinese (EPQ)

*Eysenck Personality Questionnaire* (EPQ) was devised by British psychologists Prof. Hans Jürgen Eysenck and his wife Sybil B. G. Eysenck, and its first edition was introduced in 1975. It includes four scales – Extraversion (E), Neuroticism (N), Psychoticism (P) and Lie (L), 90 items in total (Eysenck H. J., 1996). The Scale E reflects the extraversion tendency of an individual with a higher score indicating greater extraversion extent and a lower score bespeaking greater introversion extent; participants with a higher score are extrovert, restless and flexible, while those with a lower score are introvert, quiet, introspective and easy to be pessimistic. The Scale N shows the emotional stability of an individual with a higher score indicating more unstable emotion; participants with unstable emotion are subject to changing moods and excitable, while those with stable emotion are of slow and slight response and very easy to calm down. The Scale P reflects the psychopathic tendency and shows whether an individual has some psychopathic and antisocial traits of character, with a higher score embodying stronger psychopathic tendency; participants with a higher score are indifferent, solitary and hostile, while those with a lower score are obedient, considerate and cooperative. The Scale L is a validity scale and is used to test the “conceal” tendency of participants; if the score is over 70, the test is invalid.

In 1986, Professor Gong Yaoxian revised the Chinese edition EPQ for children (Gong, 1986). The revised edition is used to investigate the personality type of children aged between 7 and 15, which still includes four scales with total number of items revised to 88. The coincidence rate of these items is 87.50%-97.82% compared with those in the original EPQ, and the item contents are more suitable to the actual situation in China. Moreover, with Chinese children norm, the reliability and validity of the scale are relatively higher. Eysenck considers that the combination of Psychoticism and Neuroticism dimensions can indicates various neuroses and psychoses; therefore, participants who have higher-than-norm N and P average scores are identified as personality deviation type in this research.

#### 2.2.3 Mental Health Test (MHT)

Based on *General Anxiety Test* developed by Suzuki Kiyoshi in Japan, Prof. Zhou Bucheng, together with his colleagues from the School of Psychology and Cognitive Science, East China Normal University, introduced their revised edition entitled *Mental Health Test* (MHT), which became the standard test edition for primary and secondary school students in China (Yang et al., 2004). This test is carried out mainly in two aspects, which are anxiety-appointed objects and anxiety-led behaviors. The whole scale consists of 8 content scales and one validity scale, with 100 items in total. An integration of results derived from these 8 content scales demonstrates the general anxiety level of a student; while the result of each content scale provides evidence for the diagnosis of the major aspect concerning individual anxiety. Those 8 content scales are: A. learning anxiety (fears on examinations, failure to learn securely, concern on examination score); B. interpersonal relationship anxiety (excessive emphasis on own image, fears on interpersonal relationship, social withdrawal); C. loneliness tendency (loneliness, depression, not good at interpersonal communication, self-reclusive); D. self-accusation tendency (self-abasement, doubt on own abilities, attribution of failure to oneself); E. sensitiveness tendency (excessively sensitive, easy to be annoyed on some trifles); F. physical symptoms (emesis, insomnia, aconuresis and other overt symptoms under high anxiety); G. horror tendency (relatively severe horror on some routine matters, such as darkness); and H. impulsion tendency (extremely impulsive with relatively bad possessiveness). And “I” is validity scale (the questionnaire will be invalid if the score is 7 or above). For each item, this scale adopts a dichotomous (Yes/No) scoring system, with 1 point for each “Yes” and 0 point for each “No”. Children achieving a score higher than 65 are labeled as mentally unhealthy group, while those achieving a score lower than 65 belong to mentally healthy group. For any child achieving an individual item score higher than 8 in MHT, he or she must be provided with a targeted special guidance plan.

SPSS 16.0 is adopted to carry out statistical analysis of relevant data, which includes *t*-test, chi-square test, relevant analyses and regression analysis.

## 3. Results

### 3.1 Characteristics of Urban Left-behind Children’s Internet-Addiction

#### 3.1.1 General Characteristics of Urban Left-behind Children’s Internet-Addiction

According to the questionnaire testing standard, of all the 332 urban left-behind children tested, 36 can be categorized as the internet-addicted, comprising 10.8% of the entire urban left-behind children tested, while the remaining 296 can be categorized as the non-internet-addicted (the normal group), comprising 89.2% of the entire urban left-behind children tested. The average score of the internet-addicted urban left-behind children group reached 3.44, while the average score of the normal urban left-behind children group was 2.00.

#### 3.1.2 Respective Internet-Addictions Exhibited by Urban Left-Behind Children and Non-Left-Behind Children

Taking left-behind children as independent variables, an independent sample *t*-test was carried out both for urban left-behind children and non-left-behind children in terms of all dimensions of internet-addiction and the total scores. The result of this test indicated that between urban left-behind children and non-left-behind children, there were significant differences in the salience dimension, and no significant difference in other dimensions or the total scores (Table 1).

**Table 1:** Comparison of Internet-addiction Differences between Urban Left-behind Children and Non-left-behind Children (*M±SD*)

	Left-behind(n=332)	Non-left-behind(n=464)	*t*	*P*
Salience	2.41±0.96	2.22±0.68	-2.10*	.036
Tolerance	1.06±0.45	1.10±0.48	-1.05	.291
Compulsive Internet use	0.97±0.28	0.98±0.31	-0.30	.758
Mental Alteration	2.03±0.67	2.05±0.64	-0.32	.746
Social Comfort	1.36±0.50	1.39±0.53	-0.91	.363
Negative Outcomes	1.25±0.29	1.26±0.29	-0.42	.668
Total Score	2.16±0.68	3.44±0.56	-1.39	.163

Note.

* *p*<0.05,

** *p*<0.01, ***p<0.001.

#### 3.1.3 Gender Difference of Urban Left-Behind Children’s Internet-Addiction

According to the questionnaire results, of all the urban left-behind children tested, 298 belonged to the normal group; and 36 were internet-addicted, among whom 25 were male, with an addiction rate of 7.53%; and 11 were female, with an addiction rate of 3.31%. Given the considerable unequal numbers of students, chi-square test was adopted here in this study. The results indicated that gender difference did exist in the internet-addiction of urban left-behind children, with male addiction rate higher than that of female (Table 2).

**Table 2:** Comparison of Gender and Internet Addiction Difference of Urban Left-behind Children

	Internet-addicted Group	Normal Group	*x^2^*	*p*
Male Students	25	126	9.35**	.002
Female Students	11	170		

*Note.* * *p*<0.05,

** *p*<0.01,

***p<0.001.

### 3.2 Personality Traits of Internet-Addicted Urban Left-Behind Children 

#### 3.2.1 General Characteristics of Urban Left-behind Children’s Personality Traits

The results of statistics as per the questionnaire test standard indicated that 51 children, among the 332 urban left-behind children tested, were of personality deviation and the remaining 281 were of normal personality, accounting for 15.36% and 84.64% of the total participants respectively. In the adolescent norm, the average scores of Scale N and Scale P are 5.98 and 4.02 respectively as for male students, and as for female students, those scores are 6.08 and 3.08. In this study, the scores were 9.19 and 4.23 as for male urban left-behind children and were 10.97 and 3.81 as for female ones. However, in the personality-deviated group of the urban-behind children, the cores were much higher, being 14.19 and 8.57 as for the male students and 16.00 and 8.26 as for the female students.

#### 3.2.2 Group Difference of Urban Left-behind Children on Personality Traits

The results of the questionnaire survey indicated that 14 children, among the 36 internet-addicted urban left-behind children, and 37 children, among the 296 normal ones, were of personality deviation with a deviation rate of 38.88% and 12.5% respectively. Because of the unequal numbers of students’ distribution, chi-square test was adopted. The test results showed that in the urban left-behind children, there was a significant difference between the internet-addicted group and normal group on personality traits, and the personality deviation rate of the internet-addicted group was higher than that of the normal group (Table 3).

**Table 3:** Comparison of Internet-addiction and Personality Traits Differences of Urban Left-behind Children

	Deviated Personality	Normal Personality	*x^2^*	*p*
Internet-addicted Group	14	22	17.19***	.000
Normal Group	37	259		

*Note.* * *p*<0.05, ** *p*<0.01,

*** *p*<0.001.

#### 3.2.3 Gender Difference of Internet-Addicted Urban Left-Behind Children on Personality Traits

As shown in Table 4, among the internet-addicted group of the urban left-behind children tested, 7 male students and 7 female students were of personality deviation while the other 18 male students and 4 female students were normal. The test result indicated that gender difference did exist in the personality deviation of internet-addicted urban left-behind children, with female deviation rate higher than that of male.

**Table 4:** Comparison of Gender and Personality Traits Differences of Internet-addicted Urban Left-behind Children

	Deviated Personality	Normal Personality	*x^2^*	*P*
Male Students	7	18	4.08*	.043
Female Students	7	4		

Note.

* *p*<0.05,

** *p*<0.01, ***p<0.001.

### 3.3 Mental Health of Internet-Addicted Urban Left-Behind Children

#### 3.3.1 Group Difference of Urban Left-behind Children on Mental Health

The number of children who had mental health problems was 28. Among them, 10 belonged to internet-addicted children. Of all the 36 internet-addicted children, a mental health problem rate was 27.77%; as for the normal group comprising 296 people, 18 of them had mental health problems, with a mental health problem rate of 6.08%; the psychological problem rate of the total urban left-behind children tested (332 persons) was 8.43%. For the group of internet-addicted urban left-behind children with mental health problems (10 children), the average score was 74.10; while such score of the mentally-healthy group (304 persons), in the urban left-behind children tested, was 41.75. The average score of mental health in the total urban left-behind children (332 persons) was 44. The test results were shown that there was a difference on mental health between the two groups and the psychological problem rate of the internet-addicted group was higher than that of the normal group (Table 5).

**Table 5:** Comparison of Internet Addiction and Mental Health Difference of Urban Left-behind Children

	Mentally Unhealthy	Mentally Healthy	*x^2^*	*p*
Internet-addicted Group	10	26	19.56***	.000
Normal Group	18	278		

Note: * *p*<0.05, ** *p*<0.01,

*** *p*<0.001.

#### 3.3.2 Gender Difference of Internet-Addicted Urban Left-Behind Children on Mental Health

The number of students who had psychological problems was 7(males) & 2(females) and that of students who were mentally healthy was 18(males) & 9(females) in the internet-addicted group of the urban left-behind children, with a psychological problem rate of 28% and 18% respectively. The results figured that there was no apparent gander difference in the mental health of the internet-addicted urban left-behind children.

**Table 6:** Comparison of Gender and Mental Health Differences of Internet-addicted Urban Left-behind Children

	Mentally Unhealthy	Mentally Healthy	*x^2^*	*p*
Male Students	7	18	0.72	.394
Female Students	2	9		

### 3.4 Relationships Among Internet-Addiction, Personality Traits and Mental Health

#### 3.4.1 Research Findings of Correlations Among Internet-Addiction, Personality Traits and Mental Health

Given that this study labeled children whose average scores of neuroticism and psychoticism scales were higher than that of the norm as personality-deviated group, only neuroticism and psychoticism scales were included in correlation analysis. This study carried out correlation analysis from personality traits, as well as mental health. The results demonstrated that there were highly significant positive correlations among psychoticism, salience, tolerance, compulsive surfing of the internet, social comfort and the total score of internet-addiction; that there were highly significant positive correlations among neuroticism, salience, tolerance, compulsive surfing of the internet, change of mental state, social comfort and the total score of internet-addiction, and there was a very significant positive correlation between neuroticism and negative consequence; and that there were highly significant positive correlations among the total score of anxiety, neuroticism, psychoticism, salience, tolerance, compulsive surfing of the internet, social comfort, negative consequence and the total score of internet-addiction, and there was a very significant positive correlation between the total score of anxiety and change of mental state.

#### 3.4.2 Regression Results of Internet-addiction, Personality Traits and Mental Health

(1) Regression of Internet-addiction and Personality Traits

In order to get further understanding of the role internet-addiction plays in personality formation, based on the results derived from Table 7, taking the total scores of internet-addiction and each dimension as independent variables, and personality traits as a dependent variable, this study selected relevant typical items to carry out regression analysis, the result of which was listed in Table 7.

**Table 7:** Regression Analysis Table of Internet-addiction and Personality Traits

Independent Variable	Dependent Variable	*β*	*R^2^*	*ΔR^2^*	*F*
Salience	Psychoticism (P)	0.27	0.07	0.07	27.71***
	Neuroticism (N)	0.21	0.04	0.04	16.48***

Tolerance	Psychoticism (P)	0.32	0.10	0.10	38.31***
	Neuroticism (N)	0.30	0.09	0.09	34.37***

Compulsive Internet Use	Psychoticism (P)	0.31	0.10	0.10	37.34***
	Neuroticism (N)	0.33	0.11	0.11	42.87***

Mental Alteration	Psychoticism (P)				
	Neuroticism (N)	0.21	0.04	0.04	16.31***

Social Comfort	Psychoticism (P)	0.20	0.04	0.04	14.99***
	Neuroticism (N)	0.29	0.08	0.08	31.80***

Negative Outcomes	Psychoticism (P)				
	Neuroticism (N)	0.15	0.02	0.02	7.93**

Total Score of Internet-addiction	Psychoticism (P)	0.34	0.09	0.09	33.32***
	Neuroticism (N)	0.33	0.11	0.11	42.29***

*Note.* * *p*<0.05,

** *p*<0.01,

*** *p*<0.001.

The above statistics indicated that all dimensions of internet-addiction and their total scores had regression effects on neuroticism; and that the total scores of internet-addiction, salience, tolerance, compulsive internet use and social comfort all had regression effects on psychoticism, with the interpretation ratio of each independent variable to dependent variable ranging from 2% to 11%.

(2) Regression of Internet-Addiction and Mental Health

In order to get further understanding of the role internet-addiction plays in mental health, based on the results derived from Table 7, taking the total scores of internet-addiction and each dimension as independent variables, and mental health as a dependent variable, this study selected relevant typical items to carry out regression analysis, the result of which was demonstrated in Table 8.

**Table 8:** Regression Analysis Table of Internet Addiction and Mental Health

Independent Variable	Dependent Variable	*β*	*R^2^*	*ΔR^2^*	*F*
Salience	Total Score of Anxiety	0.20	0.04	0.04	14.33***
Tolerance	Total Score of Anxiety	0.23	0.05	0.05	19.78***
Compulsive Internet Use	Total Score of Anxiety	0.28	0.08	0.08	29.48***
Mental Alteration	Total Score of Anxiety	0.17	0.03	0.03	10.23**
Social Comfort	Total Score of Anxiety	0.23	0.05	0.05	19.53***
Negative Outcomes	Total Score of Anxiety	0.24	0.06	0.06	21.30***

Total Score of Internet-addiction	Total Score of Anxiety	0.33	0.10	0.10	44.44***

*Note.* * *p*<0.05,

** *p*<0.01,

*** *p*<0.001.

The data above show that all dimensions and total score of internet addiction had a regression effect on the total score of anxiety and the interpretation rate of each independent variable varies from 3% to 10% on the dependent variables.

## 4. Discussion

### 4.1 Analysis of Internet Addiction of Urban Left-Behind Children

According to the result of previous investigations, adolescents made up 17% of the Chinese netizens browsing the websites registered in China in 2004. The holders of junior high school degree made up 27.5% of 420 million Chinese netizens in 2010. The study had found that 10.8% of the urban left-behind children were addicted to internet. The 3.6%-11.5% of adolescents who were addicted to internet revealed by other studies (Jin, Qu, & Wang, 2010) suggested that a great number of urban left-behind children were addicted to internet.

In terms of internet-addiction salience, the urban left-behind children were more likely to get addicted to the internet than non-left-behind children. Possible reasons for this are listed as below: I. the urban left-behind children try to seek a sense of security and belongingness they lacked in the absence of their parents by surfing the internet; II. they suffer from the depression caused by inferiority, reticence, pessimism, unsociability etc. arising from the separation from their parents, and want to get relief in internet games and other fantastic novelties on the internet (Zhang, Yang, Hou et al., 2008; Pei, Zhang, & Fei, 2008). Therefore, the urban left-behind children more frequently visit and stay longer at the internet than those who stay with their parents.

The internet-addiction rate of boys was higher than that of girls among the urban left-behind children, which was in line with the previous research finding that male internet-addiction rate was higher than female’s (Su, Gao, Xiao et al., 2011). Generally speaking, in comparison with boys of the same age, girls are more mature and self-disciplined and pay more attention to information and knowledge provided by internet while many boys take internet as one of the necessities of life. In terms of disposition, boys are more likely to indulge in adventurous, exciting, novel and challenging things. And the games and sex-related information on line give them mental pleasure. Because of the anonymity of internet, boys are likely to relieve them from ill emotions by surfing internet. The major predictive variable of internet addiction lies in its function of releasing. The informative function is not the factor of the increase in internet addiction degree (Zheng, Shen et al., 2009).

### 4.2 Analysis of Personality Traits of Internet-Addicted Urban Left-Behind Children

The average level of neuroticism and psychoticism showed by urban left-behind children were all above the level of the norm. The personality deviation group of proportion was not big, but the average level of this group was far higher than the norm, with girls’ deviation rate being higher. Generally, people with high neuroticism suffer from a lack of emotional stability, and are more capricious, anxious and prone to be agitated. Meanwhile, people with high-degree psychoticism are obstinate, stubborn, solitary, unfriendly, less adaptive to external environment, lacking sympathy and concern for others. They even do something peculiar regardless of dangers. Therefore, necessary attentions shall be paid to the personality traits of urban left-behind children.

The personality deviation rate of the internet-addicted group is higher than that of the normal group. There is a study (Yong, 1999) indicating that internet-addicted teenagers get higher scores in Scale N and Scale P in comparison with the control group and they have particular personality characteristics. These characteristics are embodied as that they often show anger, depression, anxiety, impulsion and other negative emotions and behavior patterns, seek stimulus and receive less social supports in actual lives in terms of neuroticism. With respect of psychoticism, they are solitary and less adaptive to the outside world with few concerns on other persons. Only in the virtual world of internet can they find their confidence.

Among internet-addicted urban left-behind children, girls’ personality deviation rate was higher than that of boys. It was inconsistent with the previous studies. Some studies have shown that boys are more prone to be addicted to on-line games, and girls are more prone to be addicted to on-line relations (Zheng, Shen et al., 2009). A study (Huang, Qian, et al., 2006) has shown that individuals who are addicted to on-line relations show healthier and more normal traits in various tests on personality trait, emotion and cognition. Individuals with higher game-addicted tendency show more problems and pathologic propensities in the aspects mentioned above. The inconsistence with the previous studies may be cause by the limited number of samples which were not typical enough. It should be further studied for details in the future.

### 4.3 Analysis of Mental Health of Internet-Addicted Urban Left-Behind Children

The result of mental health was consistent with the comparative result of the two groups’ personality traits, and the personality deviation rate of internet-addicted group and occurrence rate of psychological problems were all higher than the normal group. Mental health, as one of the psychological and physical state of an individual, can be shown in a pattern of personality traits. And introvert people with neuroticism are more prone to suffer from mental problems (Hu & Wang, 2006). A study has shown that the internet-addicted teenagers score higher than normal ones in terms of neuroticism and psychoticism, and are more introvert, solitary, shy, and depressed (Wang, Zhang, Qi et al., 2009). Individuals with introvert and instable personalities will show ill emotions like depression and anxiety in face of dilemma, exerting impact on individual normal socialization and causing maladjustment to society. In turn, it will make them more addicted to internet.

Although females and males in internet-addicted group are addicted to different things, the left-behind children who indulge themselves in internet to seek the sense of safety and belongingness will become more introvert and unsocial. Finally, it will give rise to some problems in aspects like mental health of teenagers whose outlook on life and values have not taken shape.

### 4.4 Analysis of Relationships of Internet Addiction, Personality Traits and Mental Health

#### 4.4.1 Analysis of the Relation Between Internet-Addiction and Personality Traits of Urban Left-Behind Children

There was a positive correlation between the total scores of internet-addiction and the dimensions (salience, tolerance, compulsive surfing of the internet, and social comfort), and psychoticism. Individuals with high psychoticism show their solitary character and difficulty in adapting to external environment. Internet has created a world which can bring them sense of achievement and satisfaction, providing access to avoiding problems in real life for them. Teenagers addicted to internet often show negative emotions which can exert negative impact on teenagers’ study and life. The ill emotions include sense of aloneness, helplessness, anxiety and depression (Pang, Wu, Zeng et al.,2010; Li & Deng, 2013). Teenagers bring these emotions into life and study, forming a vicious circle. Through spending a lot of time on games, communication, and entertainment information on line, they relieve these ill emotions. Finally they will develop a compulsive addiction to internet. Teenagers’ outlooks on life and values have not been fully developed, and they have limited ability of critical thinking. The propensity of addiction makes them internalize the unhealthy on-line information and change their behaviors and characters. The result of regression analysis indicated that the total score of internet-addiction and the dimensions of salience, tolerance, compulsive surfing of the internet, and social comfort can predict psychoticism.

Psychoticism was highly positively correlated to salience, tolerance, compulsivity, social comfort and the total scores of internet addiction, and was very positively correlated to the negative consequences. Individuals with high psychoticism show ill emotions and behavioral patterns including anger, depression, sensitiveness, impulsion, weakness, etc. They relieve the negative feelings about setbacks in real life through surfing the internet, and then acquire the sense of satisfaction and achievement. Some researchers have pointed out that the individuals with personality traits characterized by psychoticism show a higher occurrence rate of internet addiction, and are prone to take internet as the best spiritual ballast (Wang & Zhao, 2007). The result of regression analysis also indicated that the dimensions and the total scores of internet addiction can predict neuroticism.

#### 4.4.2 Analysis of the Relation Between Internet Addiction and Mental Health of Urban Left-Behind Children

The total score of mental health was correlated with neuroticism, psychoticism, the total score and dimensions of internet addiction. Some studies have shown that emotional stability exerts great impact on an individual’s mental health. The more stable your emotions are, the healthier you are in terms of mentality (Zhang & He, 2008). Internet addiction also exerts impact on physical health. Surfing the internet for a long time will give rise to dyspepsia, nausea, anorexia, loss of weight etc. Meanwhile, abnormal secretion of dopamine, which is correlated with delight and excitement, will occur, followed by emotional depression and anxiety. The virtual relation established through internet is unstable and hard to make in-depth development, which goes against the development of interpersonal communication in real life. Individuals addicted to internet lack interpersonal communication skills, giving rise to weaker family ties and less communication. They are so taciturn that they frequently suffer from loneliness. Teenagers addicted to internet are highly sensitive to the surroundings, and often worry about something unreasonable and fall into fear even if nothing has happened. They show unexplainable fear even if the thing has not occurred. Teenagers will get rid of ill emotion by surfing the internet to find pleasure and satisfaction when they are unhappy with something in real life. For example, they may have poor academic performance and feel lonely, self-condemned or worried about something. The result of regression analysis also indicated that the dimensions and total scores of internet addiction can predict mental health (Y-Q. Guo & Y. Guo, 2011).

## 5. Conclusions

1) Among the urban left-behind children in China, their 10.8% internet-addiction rate is on the high side, and that the internet-addiction rate of boys is higher than that of girls. In terms of the salience of internet addiction, urban left-behind children’s occurrence rate is remarkably higher than that of those who stay with their parents.
2) In China, the personality deviation rate of the overall left-behind children is 15.36%; while the personality deviation rate of the internet-addicted urban left-behind children is 38.88%, a figure prominently higher than that of the non-addicted urban left-behind children group, with the rate among females higher than that among males.
3) The mental health problem rate of the overall urban left-behind children in China reaches 8.43%; while the rate of the internet-addicted urban left-behind children is 27.77%, a figure obviously higher than that of those urban left-behind children who are not addicted to the internet.
4) There are significant relationships among internet addiction, personality traits and mental health. The total score of internet-addiction and its related dimensions can serve as indicators of personality neuroticism, psychoticism and mental health.

Because the internet addition of urban left-behind children is a complex process, this study is only a preliminary analysis on the problem of such group, and further researches on its causes and mechanism are needed in the future.
